# Mesocosm Experiments to Quantify Predation of Mosquito Larvae by Aquatic Predators to Determine Potential of Ecological Control of Malaria Vectors in Ethiopia

**DOI:** 10.3390/ijerph18136904

**Published:** 2021-06-27

**Authors:** Beekam Kebede Olkeba, Peter L. M. Goethals, Pieter Boets, Luc Duchateau, Teshome Degefa, Kasahun Eba, Delenasaw Yewhalaw, Seid Tiku Mereta

**Affiliations:** 1 Department of Animal Sciences and Aquatic Ecology, Ghent University, Coupure Links 653, Building F, 9000 Ghent, Belgium; Peter.Goethals@UGent.be (P.L.M.G.); pieter.boets@oost-vlaanderen.be (P.B.); 2 Department of Environmental Health Science and Technology, Jimma University, Jimma P.O. Box 378, Ethiopia; kasahunebako@yahoo.com (K.E.); seidtiku@yahoo.com (S.T.M.); 3 Department of Environmental Health Science, Hawassa University, Hawassa P.O. Box 1560, Ethiopia; 4 Provincial Centre of Environmental Research, Godshuizenlaan 95, 9000 Ghent, Belgium; 5 Biometrics Research Centre, Faculty of Veterinary Medicine, Ghent University, 9820 Merelbeke, Belgium; luc.duchateau@ugent.be; 6 School of Medical Laboratory Sciences, Jimma University, Jimma P.O. Box 378, Ethiopia; teshedege@gmail.com (T.D.); delenasawye@yahoo.com (D.Y.); 7 Tropical and Infectious Diseases Research Center, Jimma University, Jimma P.O. Box 378, Ethiopia

**Keywords:** malaria, *Anopheles* larvae, predators, gut analysis, semi-field experiment, Ethiopia

## Abstract

Malaria parasites are transmitted to humans by infectious female *Anopheles* mosquitoes. Chemical-insecticide-based mosquito control has been successful in reducing the burden of malaria. However, the emergence of insecticide resistance in malaria vectors and concerns about the effect of the chemicals on the environment, human health, and non-target organisms present a need for new or alternative vector control intervention tools. Biocontrol methods using aquatic invertebrate predators have emerged as a potential alternative and additional tool to control mosquito populations. Ecological control specifically makes use of species insights for improving the physical habitat conditions of competitors and predators of vectors. A first step towards this is to gain knowledge on the predation potential of several typically present macroinvertebrates. Hence, this study aimed at (1) examining the influence of the predation of hemipterans on the number of emerging adult mosquitoes and (2) detecting *Anopheles* mosquito DNA in the gut of those predators. The prey and predators were collected from a range of water bodies located in the Gilgel Gibe watershed, southwest Ethiopia. A semi-field study was carried out using mesocosms which were constructed using plastic containers mimicking the natural aquatic habitat of immature *Anopheles* mosquitoes. Adult mosquitoes that emerged from the mesocosms were collected using a mechanical aspirator. At the end of the experiment, predators were withdrawn from the mesocosms and identified to genus level. Polymerase Chain Reaction (PCR) was employed to identify sibling species of *Anopheles gambiae* s.l. and to detect *Anopheles* mosquito DNA in the gut of the predators. Data were analysed using R software. Giant water bugs (belostomatids) were the most aggressive predators of *Anopheles* larvae, followed by backswimmers (notonectids) and water boatmen (corixids). All female *Anopheles gambiae* s.l. emerged from the mesocosms were identified as *Anopheles arabiensis*. *Anopheles arabiensis* DNA was detected in the gut content of hemipteran specimens analysed from the three families. The number of the adult mosquitoes emerging from the mesocosms was affected by the presence of predators. The findings of this study provide evidence of the potential use of aquatic macroinvertebrate predators as biocontrol agents against immature *Anopheles* mosquitoes and their potential to be considered as a component of integrated vector management for insecticide resistance and the combined restoration of aquatic ecosystems via smart ecological engineering.

## 1. Introduction

Mosquitoes are important vectors responsible for the transmission of a wide array of diseases [1]. Worldwide, there are more than 3000 species of mosquitoes, but female mosquitoes of the genus *Anopheles* are responsible for malaria transmission. There are 490 species in the genus *Anopheles*, and 70 of these are vectors of malaria. In sub-Saharan Africa, there are 140 *Anopheles* species, of which approximately 20 are known to transmit parasites to human beings [2,3]. To date, 44 species and subspecies of *Anopheles* mosquitoes have been documented in Ethiopia [4,5]. Of these, *An. arabiensis*, a member of the *An. gambiae* s.l., is the principal vector of malaria. Secondary vectors such as *An. funestus*, *An. pharoensis*, and *An. nili* occur more sporadically and with limited distribution in the country [6]. Following the World Health Organization (WHO) declaration on global malaria elimination programme in 2015 intending to make a malaria-free world by 2030 [7], the Federal Ministry of Health (FMoH) of Ethiopia developed an ambitious goal of eliminating malaria from all 565 districts by the year 2030 [8]. To achieve this goal, vector control measures remain a core intervention strategy [7].

Several interventions have been implemented by malaria-endemic countries to reduce the risk of malaria infection [9,10]. The two main vector control interventions, Long-Lasting Insecticidal Nets (LLINs) and Indoor Residual Spraying (IRS), have been successful in controlling indoor biting and resting mosquitoes [11]. Consequently, the burden of malaria has been substantially reduced in African countries, including Ethiopia [12]. However, the emergence of insecticide resistance in the major African malaria vectors *An. gambiae* and *An. arabiensis* and residual malaria transmission may compromise the current LLINs- or IRS-based interventions and thus threaten malaria elimination efforts [13,14]. Moreover, there are concerns about the effect of the insecticides on non-target organisms including mosquito predators [15,16] and the fact that they remain in the environment for decades [17]. Therefore, there is a need for an alternative vector control tool to reduce the current reliance on chemical-insecticide-based mosquito control to achieve malaria elimination by 2030 [18].

The natural regulation of mosquito larvae is an important factor in determining the survivorship of mosquito immature stages [19]. The biocontrol methods, especially those involving the use of macroinvertebrate predators as natural enemies, are recognized as environmentally friendly and are the focus of current research and control of mosquito populations [20,21]. Interestingly, the co-occurrence of immature mosquitoes and macroinvertebrates in water bodies gives a good opportunity for predatory macroinvertebrates to be used in mosquito control [22,23,24,25]. Several macroinvertebrates in the Coleoptera, Hemiptera, Odonata, and Diptera orders are known to reduce the density of mosquitoes by predating on immature stages and/or disrupting their rate of development [26,27]. Most studies have focused mainly on macroinvertebrates belonging to the order Hemiptera which forage near or below the water surface to catch their prey and play a strong role in reducing mosquito population density [21]. This raised concern regarding the possible use of macroinvertebrate predators in aquatic ecosystems as biocontrol agents against immature mosquitoes. However, to the best of our knowledge, there are no documented data from semi-field studies on the predation efficacy of aquatic macroinvertebrates on *Anopheles* mosquito immatures in Ethiopia.

A laboratory-based experimental study carried out by Eba et al. [19] in Ethiopia showed that macroinvertebrate predators from different families have significant predatory effects on *Anopheles* larvae. However, no information is available on the influence of macroinvertebrate predators on the number of emerging adult mosquitoes, which is studied here in a mesocosm environment. This study therefore aimed at (1) examining the influence of predation of hemipterans on the number of adult mosquito emergence and (2) detecting *Anopheles* mosquito DNA in the gut of those predators.

## 2. Materials and Methods

### 2.1. Predators Collection

Hemipterans were collected from a pond situated in the Gilgel Gibe watershed of southwest Ethiopia using a scoop net with a mesh size of 300 µm supported by a metal frame. Collected hemipterans were identified to family level (i.e., belostomatids, notonectids, and corixids) morphologically using standard identification keys [28]. Family-level-identified predators were put in labelled plastic containers containing water from the natural breeding habitat and covered with a net (mesh size of 1.2 mm). Afterwards, they were transported to the experimental setup in Jimma University’s compound. A few twigs of aquatic plants collected from the same habitat were placed in the containers as food and resting sites for the predators.

### 2.2. Prey Collection

*Anopheles* larvae were collected from mosquito breeding habitats found in the Gilgel Gibe watershed by dipping technique [29]. The water was collected in a mosquito-rearing enamel tray and carefully observed for the presence of *Anopheles* larvae. All larvae were sorted to genus *Anopheles* and *Culex*. Using a pipette, the *Anopheles* larvae were gently picked up based on their morphological characteristics [3], put in a bowl containing water from the same habitat, covered with a net (mesh size of 1.2 mm), and then transported to the mesocosm prepared for the study. The first-instar *Anopheles* larvae were sorted based on their size [30]. The larvae were provided with dog biscuits to forage until the study was started.

### 2.3. Study Design

**Figure 1:**
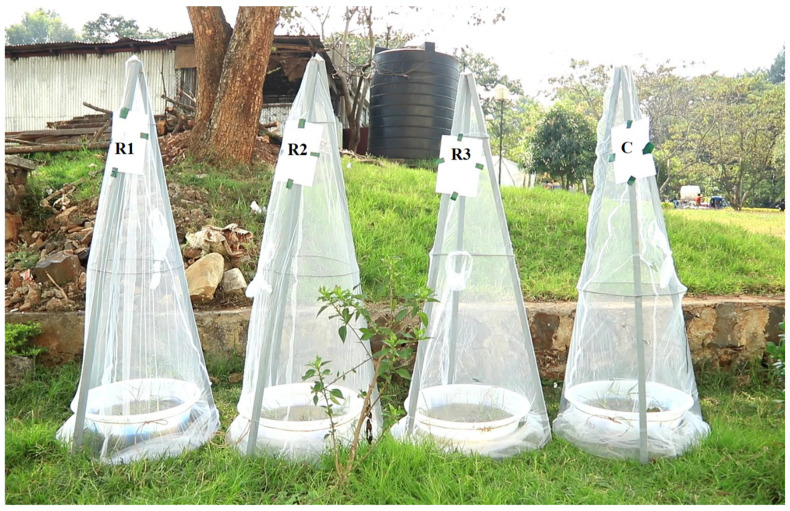
Schematic diagram of the semi-field experiment set-up (R1 = replicate 1, R2 = replicate 2, R3 = replicate 3, C = control).

Semi-natural habitats (mesocosms) were constructed using plastic containers (volume = 36 L, surface area = 750 cm^2^) mimicking the natural habitats of immature *Anopheles* mosquitoes (Figure 1). *Anopheles gambiae* s.l., the most important vector of malaria in Ethiopia, prefers habitats that are open-sunlit water pools with no or minimal vegetation [31,32,33]. Resources used to construct the mesocosms were obtained from the habitats where *Anopheles* larvae were collected. The bottom of each plastic container was covered with soil (2.5 kg). The soil texture was determined to be silty loam with a pH of 5.2, following a standard protocol [34]. A few rooted aquatic weeds were fixed to the soil at the bottom of the containers to keep them in place. The weeds belonged to six different species: *Digitaria sanguinalis*, *Echinochloa crus-galli*, *Megathyrsus maximus*, *Brachiaria ruziziensis*, *Cyperus rotundus*, and *Cyperus rigidifolius* [35]. Subsequently, water (12 L) free of mosquito larvae was added to the container. The water used in this study was stored for four days to ensure the absence of larval emergence from eggs. Identification of the weed species was carried out at the laboratory of the Department of Horticulture and Plant Sciences of Jimma University, while the soil texture and pH were determined at the laboratory of the Department of Natural Resource Management of Jimma University.

The mesocosms were randomly assigned in triplicate to four groups: the three treatment groups (with Belostomatidae, Notonectidae, or Corixidae predators) and the control (no predator). One hundred first-instar *Anopheles* larvae and ten predator individuals were introduced. The ratio of predators to larvae was 1:10 in the experiment, as described elsewhere [19], and the ratio was estimated based on the size of the bucket used for the experiment to allow the free movement of predators and prey. The experiment was carried out using 12 mesocosms in 3 blocks at a time, that is, 4 mesocosms with 3 replicates (R1, R2, and R3) and a control (C) for each predator (Figure 1). Both predators and larvae were introduced into all mesocosms in three blocks at a time. The assumption was that there were no substantial differences between the different blocks in which each predator was tested. Figure 1 depicts a photograph of the mesocosms used in the experiment for a single type of predator.

Each mesocosm of the treatment groups and control group was placed inside a conically shaped mosquito trap-net made of a metal frame and covered with a net (mesh size of 1.2 mm) to prevent any escape of mosquito vectors and to prevent invasion of the mesocosms by other species.

Adult mosquitoes that emerged from treatment and control groups were collected using a mechanical aspirator. Collected adult mosquitoes were killed using chloroform and sorted by sex, then the female mosquitoes were identified to species morphologically using taxonomic identification keys [3]. Each female *An. arabiensis* was kept in a labelled 1.5 mL Eppendorf tube over silica gel desiccant and cotton wool for further molecular analysis. The number of dead predators and larvae were recorded daily in the morning in each mesocosm. Throughout the experiment, dissolved oxygen, pH, and temperature were measured in each mesocosm every day using a Multi-Probe Meter (HQ40d Single-Input Multi-parameter Digital Meter; Hach Company, Loveland, CO, USA).

The experiment was terminated when emerging adult mosquitoes and live larvae were no longer found in the mesocosms. Following termination of the experiment, all live and dead predators in the treatment groups were withdrawn using forceps and identified to the genus level using a stereomicroscope (10×) and taxonomic identification keys [36]. Genus-level-identified predators were preserved in absolute ethanol and stored at −20 °C for further processing to detect *Anopheles* mosquito DNA in their gut.

### 2.4. Molecular Analysis of Guts

The emerged mosquitoes were identified to be *An. gambiae* s.l. morphologically. Polymerase Chain Reaction (PCR) was employed to identify sibling species of the emerged *An. gambiae* s.l. and to detect *Anopheles* mosquito DNA in the gut of the predators based on mosquito-species-specific nucleotide sequences found in the ribosomal DNA intergenic spacers [37]. The mosquito DNA extraction from the gut of the predators and from the legs and wings of *An. gambiae* s.l. was carried out using Qiagen DNeasy Blood and Tissue Kit following the manufacturer’s protocol (QIAGEN Benelux B.V.; Antwerp, Belgium). Oligonucleotide primers specific to *An. arabiensis* (5′-AAGTGTCCTTCTCCATCCTA-3′), *An. gambiae* (5′-CTGGTTTGGTCGGCACGTTT-3′), *An. amharicus* (5′AGTGTCCAATGTCTGTGAAG-3′), and universal primer (5′-GTGTGCCCCTTCCTCGATGT-3′) were used to run the multiplex PCR.

The PCR reactions were conducted in a final volume of 20 μL consisting of 0.25 μM of each primer, DreamTaq PCR master mix (Thermo Fisher Scientific, Waltham, Massachusetts, USA, containing DreamTaq DNA Polymerase, DreamTaq Green buffer, MgCl_2_, and dNTPs), and 2 μL of DNA extract. The samples were amplified in a T100^TM^ Thermal Cycler (Bio-Rad, Union Mills, Indiana, USA) with cycling conditions of 95 °C for 5 min followed by 30 cycles of denaturation at 94 °C for 30 s, annealing at 50 °C for 30 s, extension at 72 °C for 30 s, and final extension at 72 °C for 10 min. The PCR products were loaded in 1.5% agarose gel premixed with ethidium bromide stain. A marker of 100 bp ladder was also run on each gel for species identification. Following the gel electrophoresis, the PCR products were visualized under a gel documentation system. The molecular analysis using PCR was carried out at Jimma University Tropical and Infectious Diseases Research Center (TIDRC).

### 2.5. Data Analysis

Data analysis was carried out using R software (Version 3.5.2) [38]. A Kruskal-Wallis Analysis of Variance was used to investigate the differences in the number of *An. gambiae* s.l. vectors emerging from mesocosms (the experimental unit) among the three predators. A Wilcoxon post-hoc multiple comparison test was used to identify significantly different pairs. The post-hoc test was Bonferroni corrected. A Wilcoxon rank-sum test was used to compare the number of *An. gambiae* s.l. vectors that emerged from the treatment groups to those emerging from the control for each predator. The test was also used to compare the number of dead *Anopheles* larvae between treatment and control groups. The significance level (*p*-value) was set at 0.05 for all tests.

## 3. Results

### 3.1. Emergence of Adult Mosquitoes from the Mesocosms

A total of 213 female *An. gambiae* s.l. mosquitoes emerged from all treatment and control groups of the three predators (Table 1). All emerged mosquitoes were identified as *An. arabiensis*. The number of *An. arabiensis* that emerged from the mesocosms with belostomatids was lower than those emerging from mesocosms with corixids (*p* < 0.05). In addition, a lower number of *An. arabiensis* emerged from the mesocosms with notonectids compared to those emerging from mesocosms with corixids (*p* < 0.05). There was no significant difference in the number of *An. arabiensis* that emerged from mesocosms with belostomatids compared to those emerging from the mesocosms with notonectids (*p* > 0.05). A significantly lower number of *An. arabiensis* emerged from the treatment groups with belostomatids as well as the treatment groups with notonectids compared to the control (*p* < 0.05). However, the number of *An. arabiensis* that emerged from the treatment groups of corixids did not differ significantly from the control (*p* > 0.05).

Mortality of *Anopheles* larvae was observed in both treatment and control groups for each predator, but the variations were not statistically significant (*p* > 0.05) (Table 1).

### 3.2. Predators’ Gut Analysis

At the end of the experiment, a total of 69 individual hemipterans classified into 8 genera were collected from all treatment group mesocosms (Table 2). The difference between the number of the predators collected from the mesocosms at the end of the experiment and the number of predators added into the mesocosms at the beginning of the experiment is due to the difficulty of retrieving the dead bodies of the predators, especially corixids because of their size. The results of molecular analysis using PCR confirmed that both live and dead predators contained DNA from *An. arabiensis*. The DNA of *An. arabiensis* was detected in 89% of the total number of belostomatids (*Hydrocyrius*) tested. Likewise, 64% of the notonectids (*Notonecta*, *Anisops,* and *Enithares*) and 56% of corixids (*Agraptocorixa*, *Micronecta*, *Sigara,* and *Trichocorixa*) tested were positive for *An. arabiensis* DNA (Table 2).

### 3.3. Water Quality Analysis

The follow-up measurements of water quality analysis from the beginning to the end of the study showed a decrease in pH and dissolved oxygen and an increase in water temperature in the mesocosms. Briefly, the dissolved oxygen in water decreased from 8.9 ± 0.9 mg/L to 4.1 ± 1.6 mg/L, while the pH of water decreased from 7.7 ± 0.7 to 5.8 ± 0.5 in mesocosms with belostomatids. In the same way, dissolved oxygen in the water decreased from 7.8 mg/L to 4.6 mg/L, while the pH decreased from 6.5 to 5.1 in the control group of belostomatids. In mesocosms with notonectids, the dissolved oxygen and pH decreased from 9.4 ± 0.8 mg/L to 4.9 ± 1.5 mg/L, and from 6.1 ± 0.2 to 5.0 ± 0.3, respectively. Likewise, there was a decrease in the dissolved oxygen (from 7.8 mg/L to 4.9 mg/L) and pH (from 6.6 to 4.1) in the control group of notonectids.

Furthermore, in mesocosms with corixids, the dissolved oxygen decreased from 10.3 ± 0.8 mg/L to 3.4 ± 0.8 mg/L, while pH decreased from 5.8 ± 0.5 to 4.9 ± 0.1. On the other hand, there was a rise in water temperature in the mesocosms with belostomatids (from 23 ± 1.2 °C to 24.4 ± 0.1 °C), notonectids (from 23.6 ± 0.8 °C to 24.4 ± 0.3 °C), and corixids (from 22.6 ± 0.8 °C to 23.1 ± 0.1 °C).

## 4. Discussion

The findings of this study demonstrate that native macroinvertebrate predators have the potential to be used as biocontrol agents for *Anopheles* larvae for malaria vector suppression to enhance malaria elimination efforts in Ethiopia and other regions with similar eco-epidemiological settings. In this study, the number of *An. arabiensis* mosquitoes that emerged from the treatment groups was low compared to the control group for each predator family evaluated. Belostomatids were the most aggressive predators against *Anopheles* larvae compared to other predators. Similarly, the predation pressure of belostomatids on immature mosquitoes was documented in previous studies [21,25]. Notonectids were the second most aggressive predators after the belostomatids. Notonectids (*Anisops* and *Enithares*) were previously found to be important predaceous hemipterans preying on mosquito larvae, including larvae of *An. gambiae* s.l. [21,39,40,41,42]. The predation pressure of these predators could be attributed to their predation strategies for their prey. Belostomatids have the ability to turn and chase prey even at the bottom of water bodies, while notonectids demonstrate horizontal movement near or below the water surface to catch their prey [43]. In addition, notonectids show vertical movements (swiftly diving under water, frequently coming to the surface for breathing) when reversing the direction of swimming, which makes them efficient predators for *Anopheles* larvae, and making the mosquito larvae a preferred prey [43,44]. Furthermore, in our study, a lower number of *An. arabiensis* emerged from the treatment groups of corixids compared to the control group. In line with this finding, previous studies conducted under laboratory and natural conditions demonstrated that corixids have potential for the control of mosquito larvae [19,25,45,46].

Mortality of predators and prey was observed in the experiment, which could be related to changes to water quality parameters in the mesocosms. Notonectids and corixids were reported to live in a slightly polluted water body with a dissolved oxygen level of 4.5–6.4 mg/L [47]. Hence, the mortality of the predators could be due to dissolved oxygen in the water decreasing below 4 mg/L, probably as a result of the decomposition of organic matter in the mesocosms. Even slight reductions in levels of dissolved oxygen in water induce oxygen stress in aquatic organisms by depriving them of an adequate oxygen supply at the tissue level (known as hypoxia) which leads to stress or death [48]. Since aquatic organisms tolerate a small pH range (6.0–9.0), a decrease in the pH of the water in the mesocosms in our study might also be a factor for the mortality of predators and prey. In our experiment, the pH of water dropped below 6, presumably due to the pH of the soil (5.2) used in the mesocosms. Fluctuating pH or sustained pH levels outside this range physiologically stresses many aquatic organisms, and can result in death [49]. Furthermore, the death of the predators and prey could be attributed to the scarcity of food sources, as the mesocosms may not have provided food as abundantly as their natural habitat.

The detection of *An. arabiensis* DNA in the gut of live and dead predators collected from the mesocosms with belostomatids (*Hydrocyrius*), notonectids (*Notonecta, Anisops,* and *Enithares*), and corixids (*Agraptocorixa*, *Micronecta*, *Sigara,* and *Trichocorixa*) showed positive reactions to *An. arabiensis* DNA in proportions of 89.3%, 64%, and 56.3%, respectively. The lower number of *An. arabiensis* from mesocosms with predators compared to control is evidence for larval ingestion during the semi-field experiment. The presence of *An. arabiensis* DNA in the gut of dead predators, particularly notonectids and corixids, indicated that the predators died after ingesting the prey. Predators mainly affect the prey directly by consumption, but also indirectly through competition with the prey for shared food sources [50] and/or by avoidance of mosquito oviposition in sites inhabited by predators, perhaps influenced by r [51,52]. Chemicals released by predators, especially the notonectids, have been shown to repel oviposition by gravid female *Anopheles* mosquitoes [51,53].

Previous studies have reported that stream edges are refugia for immature mosquitoes during dry seasons and short rainy seasons, enabling malaria vectors to persist throughout the year in different regions of Ethiopia [54,55,56,57]. As hemipterans evaluated in the present study for their predatory effect on *Anopheles* mosquitoes are abundant and native in Ethiopia [19,24,31], rearing and introducing them to natural mosquito habitats especially during dry seasons and short rainy seasons when breeding sites are few, fixed, and findable could be an effective management tool to reduce the malaria vector population. While planning for malaria elimination programmes that incorporate larval control interventions, both dry and rainy seasons should be considered. In addition, protection of the ecological integrity of the natural aquatic ecosystems is crucial to increase the abundance and diversity of such native invertebrates to enhance their role in the biocontrol of immature mosquitoes.

The concept of rearing and introducing predators into mosquito larvae habitats as biocontrol agents against immature mosquitoes has come under discussion because successful biocontrol may depend on the mosquito prey preferences of predators in the presence of other prey. In aquatic ecosystems, suitable interventions to reduce mosquitoes must maintain a balance between the conservation of non-target organisms and the reduction of the mosquito population. Fortunately, belostomatids and notonectids have been found to reduce mosquito density in the presence of multiple prey (Chironomidae larvae, fish, fingerlings, and tadpoles) [58]. The results of this study revealed that predators belonging to families Belostomatidae and Notonectidae of Hemiptera feed on *An. arabiensis* larvae to suppress the adult mosquito population emerging from mesocosms. Hence, these predators could be used as biocontrol agents to control *Anopheles* mosquito larvae while also conserving community structures, but further studies on prey–predator relationships are required under natural conditions.

This study had some limitations. The experiment was conducted under semi-field conditions and may not indicate the effects of predation under natural conditions. Moreover, the experiment was conducted only in one place and in triplicates.

## 5. Conclusions

The results of this study revealed that the three evaluated families of Hemiptera fed on *Anopheles* larvae to suppress the adult mosquito population emerging from the mesocosms. The findings of this study suggest that aquatic macroinvertebrate predators could be used as biocontrol agents against immature mosquitoes, implying that this strategy can be considered as one component of integrated vector management strategies to manage insecticide resistance and enhance malaria elimination efforts.

Further studies are also required to determine the ability of these predators to coexist with other mosquito predators (e.g., two-prey combination) under natural conditions and with more diverse communities interacting [59].

## Figures and Tables

**Table 1:** Percentage of *An. arabiensis* mosquitoes emerged and mortality rates of *Anopheles* larvae collected from mesocosms.

Predator (Family)	Treatment Group		Control Group	
	*An. arabiensis* (Median)	Mortality Rate (Median)	*An. arabiensis*	Mortality Rate
Belostomatidae	0(0) ^a^	11 (4)	49	5
Notonectidae	6(4) ^a^	6 (6)	54	11
Corixidae	48(53) ^b^	5 (5)	62	8

Note: medians followed by different superscript letters in a column differ significantly (*p* < 0.05). Different letters indicate significant difference.

**Table 2:** The number (*n*) of predators tested for the presence of *Anopheles* mosquito DNA and the percentage (%) of the predators positive for *An. arabiensis* DNA.

Predator (Family)	Predator (Genus)	Predators (Live), *n* (%)	Predators (Dead), *n* (%)	Total, *n* (%)
Belostomatidae	*Hydrocyrius*	28 (89)	0	28 (89)
Notonectidae	*Anisops*	4 (75)	0 (0)	25 (64)
	*Enithares*	5 (40)	3 (33)	
	*Notonecta*	11 (81)	2 (50)	
Corixidae	*Agraptocorixa*	2 (100)	5 (80)	16 (56)
	*Micronecta*	0 (0)	5 (100)	
	*Sigara*	0 (0)	3 (33)	
	*Trichocorixa*	1 (0)	0 (0)	

## Data Availability

The dataset generated and/or analysed during the present study is available from the corresponding author.
